# Proximity to vulnerability

**DOI:** 10.1002/aet2.10949

**Published:** 2024-02-27

**Authors:** Mitra Sadigh

**Affiliations:** ^1^ Stony Brook Renaissance School of Medicine Stony Brook New York USA; ^2^ Nuvance Health Global Health Program Danbury Connecticut USA

With every shift in the emergency department (ED), I am reminded that each of us is only one or two steps from being extremely vulnerable.

My difficult moments have not been as colored by patient stories as by their stories being lost and misconstrued; spun and respun to maintain distance between staff for whom it's another “day on the job” and patient for whom it may be the worst day or era of their life. The protective barrier transforming an individual in need of help into a problem to be dealt with.

*My difficult moments have not been as colored by patient stories as by the ways that another “day on the job” for health care workers can easily become either the worst day or worst era of their life. Sometimes that protective barrier can make us feel more safe*.

It wasn't the woman who arrived obtunded after ingesting an unknown substance in an attempt to end her life, but the disgruntled complaints that she not only needed resuscitation, but had also “sh*t herself.” The sighs of exasperation when she soiled herself again.

*It wasn't the process of uncovering what medication had been overingested, but watching the nurse meticulously wipe the dried, pressed fecal matter from the completely unconscious patient who hours ago had decided this life was not worth living*.

It wasn't the survivor of gun violence who repeatedly screamed in pain when the leg the bullet had lodged in was poked and prodded. It was staff rolling their eyes as she continued to vocalize and emote. The disbelief that she could be in so much pain against the belief that she shouldn't have been “out in the streets.”

*It wasn't seeing a leg swell up with such tension that it might burst, but watching a resident rush out of the room in tears with the patient's scratch marks across her abdomen*.

It wasn't the teenage survivor of sex trafficking who was brought to the ED against her will because she lacked a safe place to sleep. It was hearing the passing of her story from EMS to nursing to residents about how “difficult” she was and “good luck dealing with her.” That she had “probably spent every night with a new man, ‘getting some.’”

*It wasn't the physician returning disappointed after being unable to appease a teenage survivor of sex trafficking. It was hearing her say that the patient had called her a “bitch” and watching her remove herself from the care team*.

I ask you to reflect with me. What is more frightening?

The “agitated” patient who might raise their voice and threaten or losing the ability to recognize a desperately frightened human staring back?

*Losing compassion for a desperately scared human staring back or being another survivor of workplace violence*?^1^

Resuscitating someone who has attempted to end their life or being comfortable publicly humiliating them in their most vulnerable moment as they are being pulled back from the brink of death?

*Expressing momentary frustration after repeatedly cleaning feces from an unconscious patient post–suicide attempt or internally holding endless resuscitations internally to the point of becoming another healthcare worker suicide statistic*?^2^, ^3^

Witnessing someone endure the pain of a gunshot or being a person who assumes that others deserve the violence they suffer?

*Distancing from patients by assuming people deserve what they suffer or absorbing the reality of senseless tragedy, battling PTSD from the countless victims of gun violence heard screaming down the ED halls*?^4^

Absorbing the tragedy of a teenage survivor of sex trafficking or failing to recognize that she is just a child and that the police surrounding her room are scaring her?

*Misrepresenting a survivor of sex trafficking or reaching an impasse where only substance alleviates the feeling of hard work going without appreciation or impact*?^5^

In the ED, we cannot prevent life's grief from suffocating someone's will to live any more than we can prevent the landing point of a bullet or unspeakable harm to a child. We cannot prevent tragedy from making our days feel heavy any more than seeing yet another bullet wound or teenager who never had a chance at childhood. But we can do more to protect ourselves, our patients, and our passion for this work.

Recognizing that health care training alone is inadequate preparation for managing the challenges of the ED environment, we must expand our toolkit. We can dive deeper into our internal landscape to learn to build resilience^6^; better manage our biases^7^, ^8^, ^9^; equip ourselves with more intentional coping strategies^10^; and turn to mental health experts to help us reform our support systems. We can employ methods of nonviolent communication, self‐awareness, and self‐regulation that are utilized by others who work in emotionally charged settings. We can refresh our perspective by creating a standardized definition of patient‐centered care^11^ and taking steps to protect patients from further harm while in the ED.^12^, ^13^, ^14^, ^15^, ^16^ We can improve collective support by integrating regular group debriefing^17^ and more robust support in the workplace.^18^

We can commit to cultivating a beginner's mind with patients, approaching with curiosity rather than assumption or shut down. We could pause before reacting to a screaming patient and ask what is causing the patient to scream. Pain? Fear? Adrenaline? Overwhelm? Do they need pain medication? A hand on the shoulder? A few minutes alone?

*We can commit to cultivating a beginner's mind with ourselves, taking a moment to pause before reacting and asking the pertinent question of what we need at that moment. A snack*? *Three deep breaths*? *Positive affirmation*? *Someone else to see this patient*?

We can call on the strengths of our interdisciplinary teams, from chaplains who beautifully model holding emotional space and navigating tender moments to social workers who are champions of connecting patients with pertinent resources. We can speak up about the current overflowing and understaffed state of EDs, calling for new roles like patient advocates.^19^, ^20^ Rather than grinning and bearing, we can use our collective voice to share our struggles on greater platforms and push for deeply needed change. Rather than becoming disullusioned with the barriers in the care we provide, we can advocate for our patients more broadly. We can collectively work towards evolving ourselves, our work, and the ED environment in ways that better serve us and our patients.

## CONFLICT OF INTEREST STATEMENT

The author declares no conflicts of interest.
